# Deconstructing Barisanus’ medieval casting technology based on digital twins

**DOI:** 10.1038/s41598-025-91168-9

**Published:** 2025-03-03

**Authors:** Bastian Asmus, Martin Fera, Marianne Mödlinger

**Affiliations:** 1 IAW, Abt. Urgeschichtliche Archäologie, Albert-Ludwig Universität Freiburg, Belfortstrasse 22, 79098 Freiburg, Germany; 2 Labor für Archäometallurgie, Oberer Zirkel 8, 79341 Kenzingen, Germany; 3 Institut Für Urgeschichte Und Historische Archäologie, Universität Wien, Franz-Klein-Gasse 1, 1190 Vienna, Austria; 4 IMAREAL, Paris Lodron University Salzburg, Körnermarkt 13, 3500 Krems/Donau, Austria

**Keywords:** Monumental bronzes, Barisanus of Trani, Solid shrinkage, Medieval metal casting, Digital twins, Archaeology, Cultural evolution

## Abstract

The Italian 12th-century copper alloy doors of Barisanus have long been the subject of art historical studies and have recently been fully documented photogrammetrically and archaeometrically. In addition, digital twins of the three Barisanus doors in Ravello, Trani and Monreale have been produced and made available in open access for further research. These digital twins, together with the orthophotos produced, allow precise and repeatable measurements of the different metal parts of the doors, such as panels and frieze elements. This, and by taking into account the solid shrinkage of metals during the casting process, allowed for the reconstruction of the production sequence for each of the 20 single motif panels present on all three doors, and, finally, also for the establishment of the production sequence of the three doors as a whole—heavily discussed in art history research—with rather astonishing results. Moreover, bronze casting techniques used and the use of moulds and models are discussed, painting a more detailed picture that goes beyond the often purported dichotomy of lost wax vs. sand casting.

## Introduction

The workshop of the artist Barisanus of Trani can be considered one of the most active and well-known workshops for the production of copper alloy objects, in particular monumental objects, in the High Middle Ages (1000–1300 CE). Being active in the twelfth century in southern Italy, the artist and his workshop are responsible for at least four metal doors, of which three are still preserved today: the main doors of the cathedrals from Trani and Ravello, as well as the side door of Monreale. Only the door from Bari is not preserved anymore^1^.

As their predecessors, all the Barisanus doors consist of a wooden base onto which single, relatively flat metal panels were nailed onto. The gaps between the plates were covered with frame elements, which again were covered with decorated knobs where their ends meet. The panels do not contain any inlay; they depict various saints and characters from the New Testament in flat relief.

The door from Ravello is dated according to an inscription on the door to 1179, whereas the Trani door lacks any indication of when it was created completely. A similar situation can be observed in the case of the door from Monreale. It can be assumed that Barisanus constructed his door on the northern facade before Bonanno created the main door of the cathedral on the western facade. It can therefore be concluded that the production of the door took place between the foundation of the cathedral by King William II of Sicily (1166–1189) in 1174 and 1186. As Barisanus is said to be from Trani, it would be reasonable to assume that he made his first door there, or at least in the vicinity (Bari). However, this has not yet been proven. Until today, the chronological sequence of Barisanus’ works and the manner of production, in particular the question of whether models (stamps) were used in the production of copper alloy objects, remain a matter of dispute in the field of art history.

During an international project funded by the Austrian FWF, recently about 30 copper alloy doors of the eleventh-twelfth century were fully documented and chemically analysed for the very first time. In the scope of this project, also the three doors attributed to Barisanus of Trani were fully documented. The orthophotos produced allowed for the first time the reconstruction of the chronological order of the manufacture of the doors, and, moreover, to shed light on the casting technology and workshop organisation of the artisan(s).

In the following, the principles of different moulding processes known in the twelfth century and the process of solidification, shrinkage and distortion during and after the casting of copper alloys are described.

### Moulding processes in general

Before we launch into the detailed and highly selective study of the traces left by the casting processes, it is imperative to gain a fundamental understanding of the terminology and techniques associated with casting:

- Lost wax: the wax model is lost during the preparation of the mould
- Lost mould: the mould is lost upon retrieving the cast (e.g. investment or sand casting)
- Lost model: the model is lost during the moulding process (e.g. lost wax or lost foam)
- Permanent model/pattern: the model can be re-used to produce another mould
- Sand (moulded) casting: the moulding material is some sort of sand
- Permanent moulds: ceramic/stone moulds, metal dies, or similar that can be reused for other castings

In this respect it might be more helpful to differentiate the casting techniques based upon the retention of the model and/or the mould, rather than the moulding material itself, even though a unifying classification of archaeological moulding materials has not yet been established. This is even more important as we cannot prove which moulding material went into casting flasks. They may also be used successfully with moulding clay or plaster investment moulds. Moulding sand with naturally occurring clay as a binder represents one end of the spectrum, while (sandy) clay or loam forms the other end, creating a continuum between these two essential ingredients for producing casting moulds. This may illustrate why the current inherently accepted classification is not suited for a deeper understanding of ancient casting practices.

A metal casting mould can be produced in two ways: by mechanical deformation of the moulding material or by melting out a model. This model can be produced by casting in an auxiliary mould of any kind, or by mechanical work in a permanent mould. It can also be made individually. For mediaeval times there is no archaeological record for auxiliary moulds^2^. Art technological literature regularly cites Roman era bronze sculpture and casting technology often based on^3^ or^4^, and an unbroken technological tradition is implicitly or explicitly assumed.

There is one alleged piece of a plaster mould associated with the casting of the Wolf doors from Aachen cathedral^5^, however it was lost during WWII. Further it cannot currently be ascertained as to whether it is a plaster copy of a clay mould or an original plaster mould, because large parts of the documentation of the excavations at Aachen were lost, too^6,7^. However, it is possible to reproduce wax models using techniques that do not leave archaeological traces, making the absence of auxiliary moulds less surprising.

The mould material can be compacted over a model, as for example in modern sand moulding, or said pattern is used as a stamp and pressed into a more or less plastic mould material. The latter process, although possible, is not used nowadays in hand moulding where casting is concerned. In both cases though, the mould consists of at least two mould halves, which are joined together before the casting process.

The method of making the mould and model has considerable implications in terms of expected shrinkage; for instance, a wax model cast from liquid wax will be smaller than a wax model made by pressing wax into a permanent wooden mould. Technically there are **three modes of production of mould and model which are feasible**, where option 2 and 3 are only differing in the choice of moulding material and its mode of application:

1. **Intermediate/auxiliary mould to reproduce wax models, as it is done today**
  - a lost mould, e.g. from fine, plastic clay, not mechanically resilient moulds for use with liquid wax
  - a permanent mould for making a wax model, e.g. from wood, mechanically resilient moulds to make the model by solid forming
2. **Stamp to make an impression in the plastic mould material**
  - an original model (wood, plaster,…) is used
  - an original wax model that is used as stamp
  - an off cast is used as stamp
3. **Permanent model that is used to make a lost mould**
  - an original model (wood, plaster,…) is used
  - an original wax model that is used as stamp
  - an off cast that is used as stamp

How did Barisanus cast the single metal elements for his doors? Is the sand mould process, aside from its technical feasibility, a possible contender as a production process for the doors of Barisanus? Buccolieri and colleagues entertain this notion^8,9^, however their interpretation falls short on account of several aspects. It is necessary to address these issues in this context. We will have a brief look at the essential technical prerequisites on the one hand, and on the other hand at the historical and archaeological record for traces of this casting process.

The early twelfth century *schedula diversarum artium* by Theophilus Presbyter^10^, knowledgeable in all contemporary metallurgical techniques, does not mention sand casting at all, but describes the lost-wax casting in two chapters: the making of the censer and the casting of a bell^11,12^. In the western hemisphere, the sand mould process can be confirmed as a known method for the casting of smaller and/or simple objects, such as reliefs or epitaphs from the mid sixteenth century onwards. Biringuccio’s treatise of 1540^13^ is among the earliest sources to mention powders and frames for casting and it appears that at least north of the Alps the sand casting method was not used before that time. However, if we accept Dines’^14^ hypothesis and Theophilus Presbyter is not the famous goldsmith Roger of Helmarshausen, as Ilg^15^ and later Freise^16^ suggested, but several authors and collators, there are relations to Lombardy, or at least Italy, by two of these authors: the first calling himself Lumbardicus; Dines suggests the sources of the recipes in the first book of the *schedula* to be of Byzantine origin, with Lombardy and Salerno being rather probable places of origin for these texts. The second author calls himself Northungus; he might be identical with Northungus of St. Michael in Hildesheim, famous physician and encyclopaedist with a strong interest in the medical works of Salerno. He would be the author or collator of the third book, dealing with all the metal trades^14^.

In the Islamic world, the situation appears different. The 1974 translation of *The Book of Knowledge of Ingenious Mechanical Devices* by al-Jazari suggests that by the twelfth century, closed mould boxes were being used for casting in green sand^17^. In foundry terms green sand is sand that can be used in its humid state. Moreover, the text implies that this technique had been in use for some time. Notably, the reference is made in the context of manufacturing large brass doors. However, the section discussing sand casting mentions only a “casting box” and provides no further details. It also does not explain how Hill^17^ arrived at his somewhat speculative translation. There is no explicit mention of green sand or a second casting box. Instead, it seems the casting process described may have taken place in an open-hearth mould. In the following passages, al-Jazari provides much more detailed descriptions of the lost-wax casting technique. Therefore, it seems highly unlikely that the text is describing a true sand-casting process. Nonetheless, this passage warrants a critical re-translation to clarify its meaning.

**Fig. 1:**
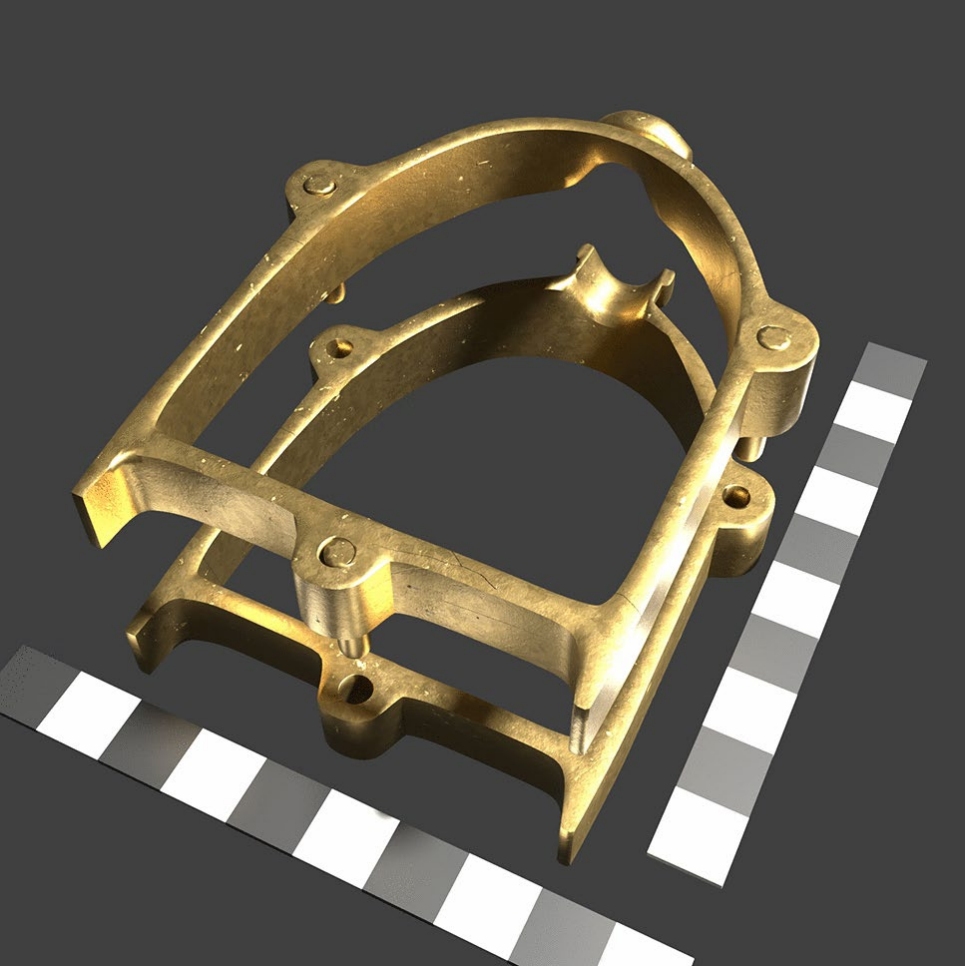
3D reconstruction of one of the Tiberias casting flasks, which are very small, and not suitable for casting door panels. Scale bar: 10 cm. Image: 2024 B. Asmus.

Susan La Niece^18^ has recently provided an insightful overview in the Islamic world, where she presented casting flasks, also referred to as moulding boxes. These are necessary for sand casting to encase the compacted sand during casting. They are dated to the eleventh-twelfth century^19^. Upon closer inspection, these are real casting flasks^20^ but are very small. There are two sets: The larger one possesses the dimensions of 13 × 8 × 1.3 cm (Fig. 1**)**. The only two other surviving casting flasks of the Islamic world are unfortunately undated finds^18^.

The key points of the sand moulding process would require a refractory sand held together by a binding agent compacted into a top and bottom casting flask: the drag and cope, which can be separated and precisely reassembled prior to casting. Casting flasks for the making of the Trani door panels would need to be of considerable dimensions, as the panels have a mean size of 32 × 40 cm. However, before discussing the hypothetical sizes of casting flasks necessary to cast panels, it seems important to revisit the reasons for the assumptions that sand moulding was used in the production of the door panels of Barisanus’ doors. Table 1 presents the hallmarks of a sand-moulded cast object compared to an object cast in a bivalve clay mould:

**Table 1:** Comparison of the traces left by the sand moulding process compared to a two-part loam moulding process.

Hallmark	Sand moulding process	Two part loam mould
Flash-line along the parting line	Yes	Yes
Little/no undercut pieces for removal of model	Yes	Yes
Ingate and gating system cut into moulding material	Yes	Yes
Casting flasks	Yes	No

When comparing the hallmarks of both processes it becomes clear that they cannot be differentiated on grounds of their appearance, unless we are looking at bronzes cast much later in the eighteenth century, where the piece mould sand process is fully established, because it leaves unmistakable traces on the cast objects behind. In the mediaeval period and before they can only be proven if casting flasks are found in connection with the examined objects.

There is much more in casting technology than just the dichotomy between lost wax and the sand moulding process, and many misunderstandings may be a result from the inadequate categorisation of these two processes: Where the lost wax is classified based on the presence/destruction of the model, the sand moulding process is classified based upon its moulding material. This classification is mostly valid for industrial or at least modern times, and is less applicable to earlier casting processes, which were characterised by a much greater variety of technological choices and a lack of scientific, and thus resulting less normative, approaches. In its place, workshop traditions based on experience and skills evolved to ensure a measure of process reliability.

### Solidification, shrinkage and distortion

**Fig. 2:**
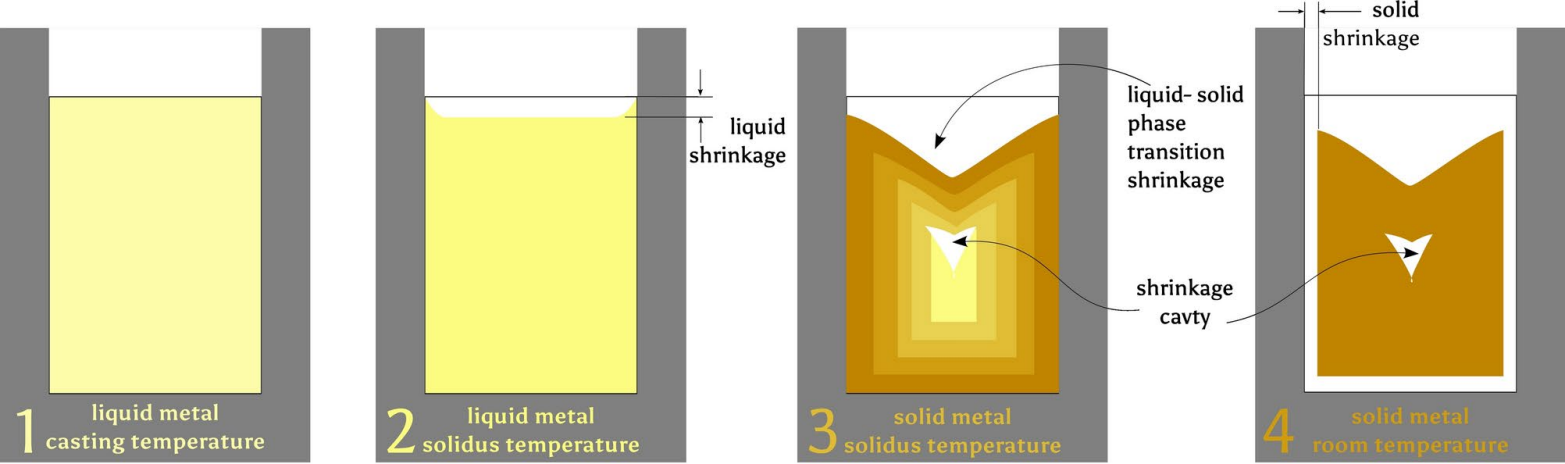
The principal stages of solidification. When metal solidifies it cools in successively from the outside to the inside, thereby decreasing its volume. If the cooling gradient is too steep, a shrinkage cavity may form. Image: 2024 B. Asmus.

Metal casting involves a phase transformation from the liquid state to a solid state (Fig. 2). This solidification process involves several stages. There are three processes at work: liquid shrinkage, shrinkage during phase transition, and solid shrinkage. Shrinkage values are generally expressed as **linear shrinkage**, that is the shrinkage along one dimension. For archaeologically relevant metals the metal solidification coincides with volume reduction. The processes are explained in the following sections.

#### Liquid shrinkage and shrinkage during phase transition

When liquid metal cools down to the solidification temperature there is a small change in volume as it contracts. On top of that there is the reduction of volume from the liquid to the solid state of matter. Since this is concerned with the still liquid state, this can be compensated by a properly designed gating system and also—intentionally or accidentally—the presence of feeders, to supply enough liquid metal to compensate for this reduction of volume (Fig. 2). Up until the casting of cannons^13^, until the turn of the 15th to sixteenth century, casting cups or funnels unintentionally acted as feeders by supplying additional liquid metal during solidification, compensating for volume reduction. Its effect can be seen in the usually funnel shaped depression in the casting cup. These shrinkages are responsible for a number of casting defects, but shall not be of concern for the purposes of this paper. They are mentioned only to identify the principal volume reduction reactions.

#### Solid shrinkage

For the purposes of this paper the solid state shrinkage is the most important shrinkage type; it occurs as the material cools from solidification to ambient temperature. This process cannot be compensated for, as it is a material property of the metal used for casting. In other words, the dimensions of the mould are always larger than the dimensions of the cast object in it. This has many implications for the study of cast objects in our history, as it provides us a tool to link cast objects and casting models, and further enables us to establish production chronologies based on simple measurements. This reduction in volume can only be accounted for if the model is designed to accommodate the shrinkage of the particular metal that is being cast. Different metals and alloys possess different shrinkage values. The values for bronzes and brasses are in the area of 1.1–1.8%^21^. Solid shrinkage can also be observed in beeswax. Models should be made larger if accurate dimensions after casting are essential. For prehistoric and most of the historical periods, this does not seem to have played a particularly important role, and it is even questionable if it was recognised:. A source from the twelfth century reveals that the concept of metal contraction was not acknowledged at the time: in his chapter on bells, Theophilus Presbyter writes that the core needed to be extracted quickly because it swelled during cooling. In reality, Theophilus misinterpreted the reason for developing cracks after casting, attributing it to a swelling core rather than the metal shrinking around a core, which could contract as much as the metal^11^. The situation becomes even more complex if we also take into account the shrinkage of the other materials that are used in the casting process, such as wax and clay.

#### An approximation of beeswax shrinkage values

**Fig. 3:**
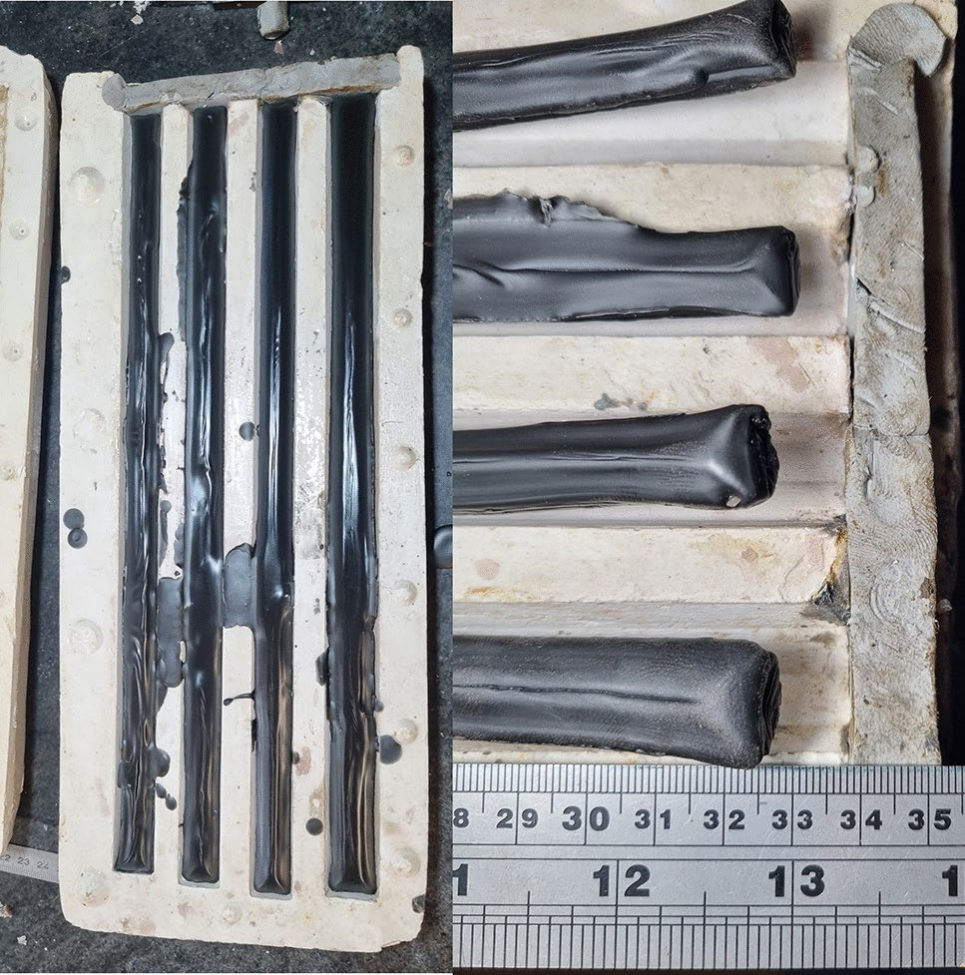
When cast in open moulds beeswax shrinkage experiments yielded overall linear shrinkage values of up to 3%. Left: wax was cast in an open mould. Right: The dramatic shrinkage can be seen with the naked eye. Note that this beeswax is dyed black with soot to enhance the visibility of the surface relief. Table 2 provides the relevant measurements. Image: 2024 B. Asmus.

There are no beeswax shrinkage values in the literature. Some preliminary experiments with beeswax yielded linear shrinkage values of up to 3% (Fig. 3 and Table 2). This value may of course also change by introducing various additives, such as tree resins, or animal fats, but is beyond the scope of this paper. As much as the liquid shrinkage can affect the cast object in metal casting, it does so in the manufacture of wax models when working with beeswax. As laid out above, these negative effects can be mitigated by properly designing a gating system. In wax model production the mode of wax application can have a similar effect and is corroborated by our brief experiments with open and closed moulds (Table 2).

**Table 2:** Measurements of cast wax rods at room temperature (9 °C).

Cold wax length	Calculated Shrinkage	Casting temperature	Room temperature	Mould type	Mould was	measured volume
in cm	in percent	in °C	in °C		cast	loss in ml
33.0	2.5	70.0	9.0	open hearth mould	horizontally	n.a
32.8	3.1	70.0	9.0	open hearth mould	horizontally	n.a
32.7	3.4	70.0	9.0	open hearth mould	horizontally	n.a
32.8	3.1	70.0	9.0	open hearth mould	horizontally	n.a
33.2	1.9	63.0	9.0	closed mould	vertically	see caption
33.5	1.2	70.0	9.0	closed mould	vertically	9.0

Mould length 33.85 mm. Casting temperature is 70 °C in all but one case. Experiment no 6 no volume loss was observed, because wax was cast at solidus temperature of 63 °C of the experiments beeswax and produced very bad cast objects with numerous cold shuts.

When beeswax was cast in open moulds, the overall linear shrinkage reached up to 3.2%. In this method, it is not possible to separate liquid shrinkage from solid shrinkage. In contrast, casting in closed moulds showed a linear solid shrinkage of approximately 1.2–1.5%. During the liquid-to-solid phase transition, a wax rod initially containing 104 ml of liquid beeswax experienced a volume reduction of 9 ml, corresponding to a linear liquid shrinkage of 2.1% or a volumetric shrinkage of 8.6%.

#### Drying shrinkage of moulds

During the drying process of moulding loams, or more generally, clay-based moulding materials, shrinkage is also encountered in several stages and usually coincides with the various stages of water loss^22,23^. Drying shrinkage of moulding materials may add an additional layer of dimensional change that must be considered alongside solidification shrinkage when interpreting archaeological cast objects. Currently there are no studies examining the influence of this type of shrinkage and a large database of properly characterised historical casting material properties needs to be established to be able to assess its exact influence.

#### The role of shrinkages

**Fig. 4:**
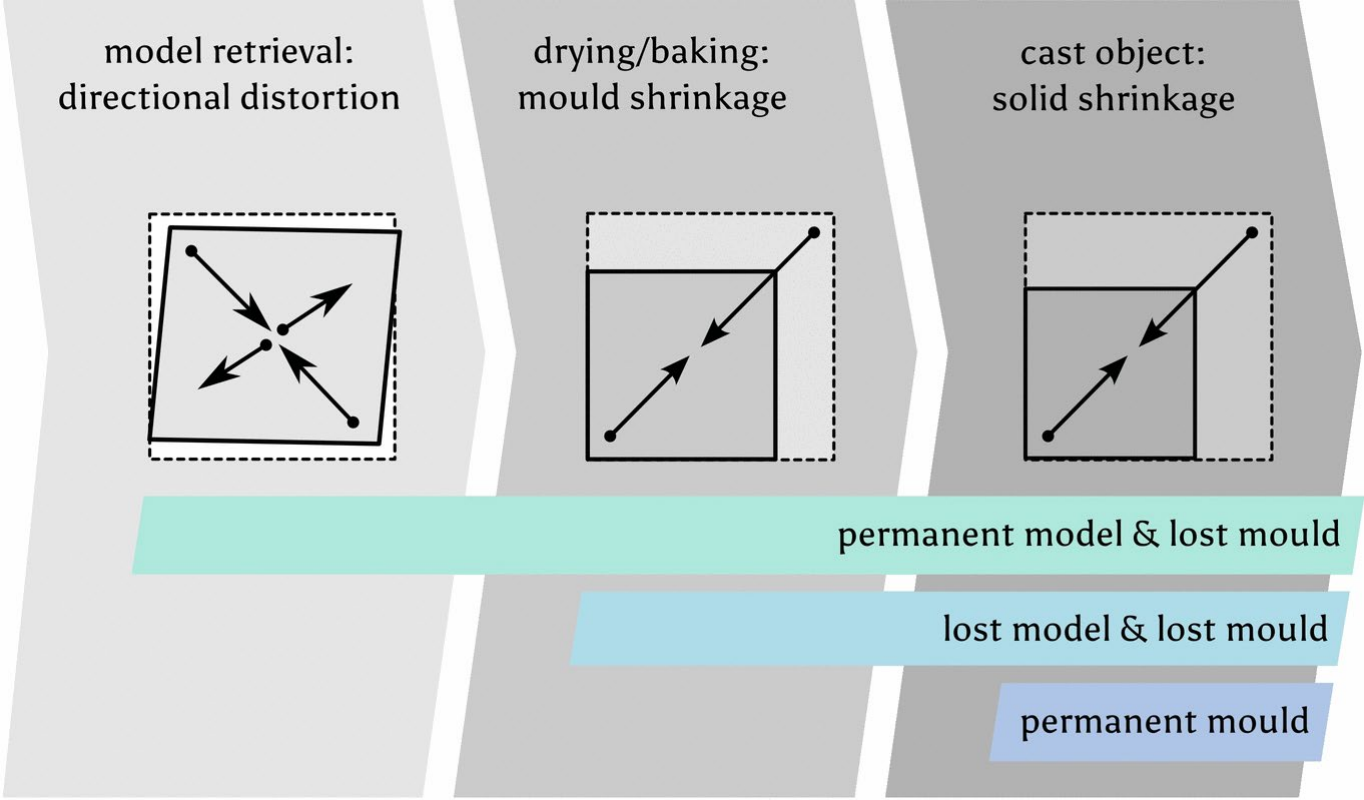
This flowchart displays the principal steps of any mould making that uses lost moulds, resulting in **one casting generation**. It highlights those factors that contribute towards overall shrinkage and distortion of a cast object compared to its model. It is true for most casting operations in prehistoric and historical contexts and independent of the materials being used. Image: 2024 B. Asmus.

Figure 4 schematically illustrates the process chain of mould making and casting, highlighting factors that contribute towards shrinkage and possibly also to unidirectional dimensional changes. We need to understand that the solid shrinkage is a physical property of the medium that is cast and cannot be avoided. The shrinkage of the mould material can theoretically be avoided by adding enough temper, however, in practice, it is hardly ever fully achieved^11^. These two factors are true for the casting of metals as well as for casting wax for the production of casting models. A third factor for dimensional change has to do with the retrieval of the model, and it is true for permanent models that were used to create a mould, as well as it is true for casting models that were made by using a permanent mould. In the first case, a permanent model may be pulled incorrectly from the still soft mould material and change the dimensional accuracy of the mould; in this case the mould cavity is larger than the model in one direction, resulting in a slightly distorted mould. The second case addresses the replication of wax models in a mould. Two principal methods are used to replicate an original model or existing cast: pouring liquid wax into a mould or pressing warm plastic wax into a mould or die. When liquid wax is poured into a mould, such as one made from soft clay, solid shrinkage must be accounted for as the wax cools and solidifies, resulting in a noticeably smaller model due to the shrinkage of beeswax. On the other hand, pressing warm plastic wax into a permanent mould, like one made of wood, typically results in minimal or no shrinkage. However, distortion can occur during the de-moulding process, especially while the wax is still warm and pliable, leading to unidirectional deformation as it is removed.

Acknowledging and recognising these issues is essential when examining archaeological objects, as they provide crucial insights into the reconstruction of the manufacturing processes used in their creation. Identifying signs of shrinkage or unidirectional distortion can help archaeologists and researchers infer the methods and materials employed, enhancing our understanding of historical craftsmanship and production techniques. The cumulative dimensional change in some cases will usually exceed the shrinkage factor of the metal used in subsequent casting steps.

## Methods

The methodological approach on which our interpretation is based has already been outlined in^1^ for the photographic documentation and production of both orthophotos and 3D surface-models. Also, the chemical composition of the different metal elements of the three doors was discussed in the same publication; the results are made available in an online-repository (Links 1–3). The data sets, utilised for further analysis consist of a true orthophoto of each door with a spatial resolution of 0.2 mm/px and a Digital Surface Model (DSM) with the same resolution, handled and visualised with relief visualisation tools in a geoinformation system (GIS).

### Measurements on orthophotos

**Fig. 5:**
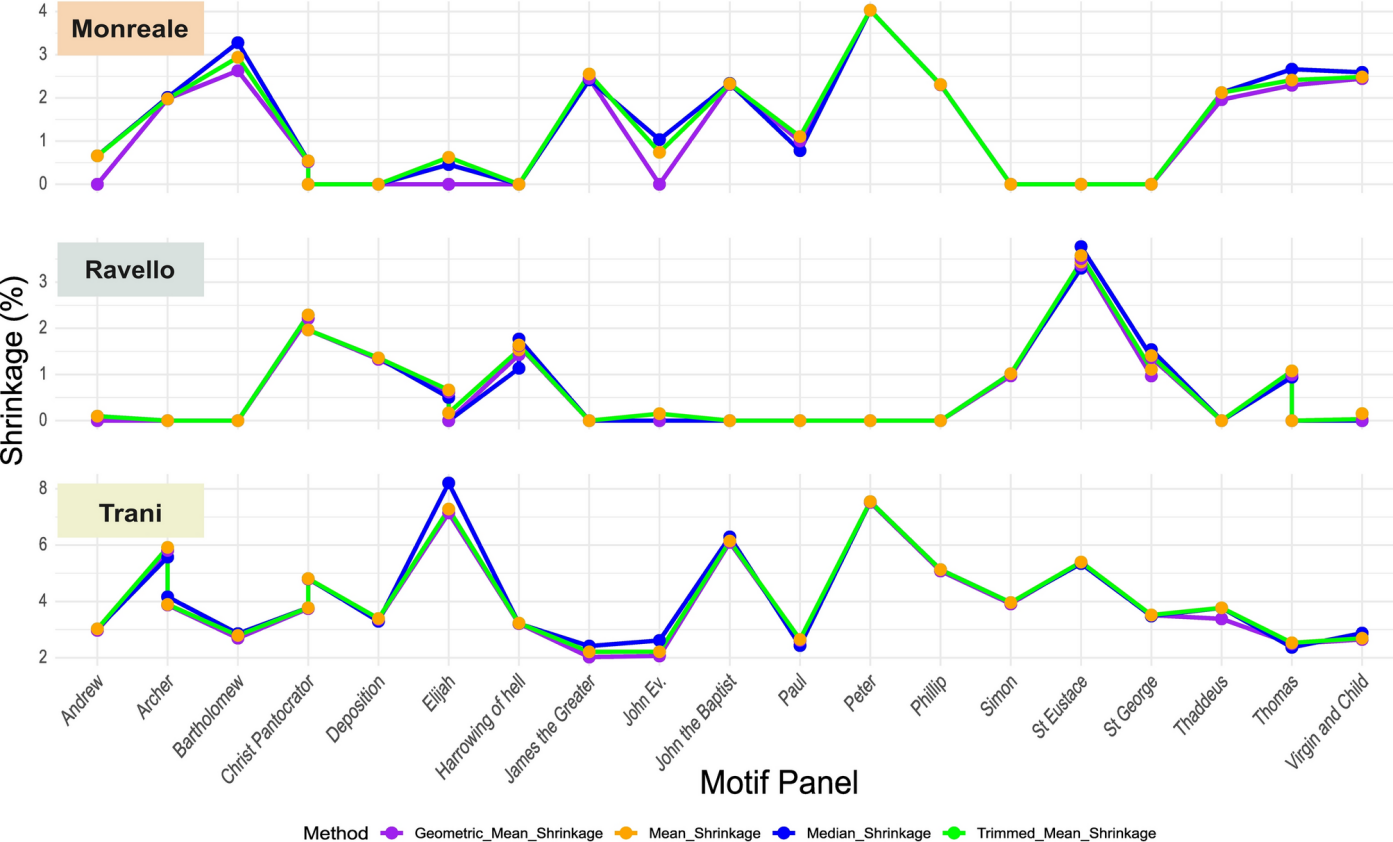
Shrinkage trends across motifs by method and door. A comparison of methods building an average value of the change in dimension based on the measurement taken from the panels.

During repetitive measurements of the same distance on the same panel, a measurement inaccuracy of ± 0.2 mm could be determined. This was used to calculate the propagated error, which is relevant for the calculated shrinkage values. Not in all cases it was feasible to take three measurements on all panels. To retain comparability across all panels displaying the same motif, it was decided to use a minimum of two perpendicular measurements per panel. This was done to record the dimensional change in the x and y direction. In a few cases three of or even four measurements could be taken. In order not to waste these measurements a statistical analysis was undertaken and evaluated how this impacted the overall classification of calculated shrinkages. We tested the mean, trimmed mean, geometric mean and the median. The overall result is presented in Fig. 5.

It shows that the median reacted too sensitive with extreme values and the geometric mean did not under-represent the low values. For this reason, the mean was used as a method to condense the measurements into a single value that is thought to represent the linear shrinkage across these panels.

## Results and discussion

The art historical approach has so far put forward the hypothesis that at least four doors can be attributed to Barisanus of Trani. Of the existence of a fourth door, that of Bari, we do only know because of a surviving early nineteenth century drawing^1^. We do not know if Barisanus was the responsible artisan for all of these doors, nor do we know as to whether he was the sculptor, the artisan, or the founder, or in which manner the production of these doors was organised. However, it is a fact that the doors of Ravello, Trani, and Monreale possess motif panels of a design so similar that some sort of copying or reproduction of these took place during the manufacture of said doors. This observation, at the end, permits us to establish a chronology of the production of these three doors.

### Motif panels

This section examines the figurative reliefs on single door panels from Trani, Monreale, and Ravello, focusing on their production methods, shared motifs, and casting processes. The frames and friezes, as well as their composition is discussed in the section below. The following questions were raised:

1. Are similar motif panels made from the same model?
2. Are they actually similar, or how similar are they?
3. What is the reason for the blurred appearance of the motifs?
4. Is it possible to reconstruct the casting method of these panels?

**Fig. 6:**
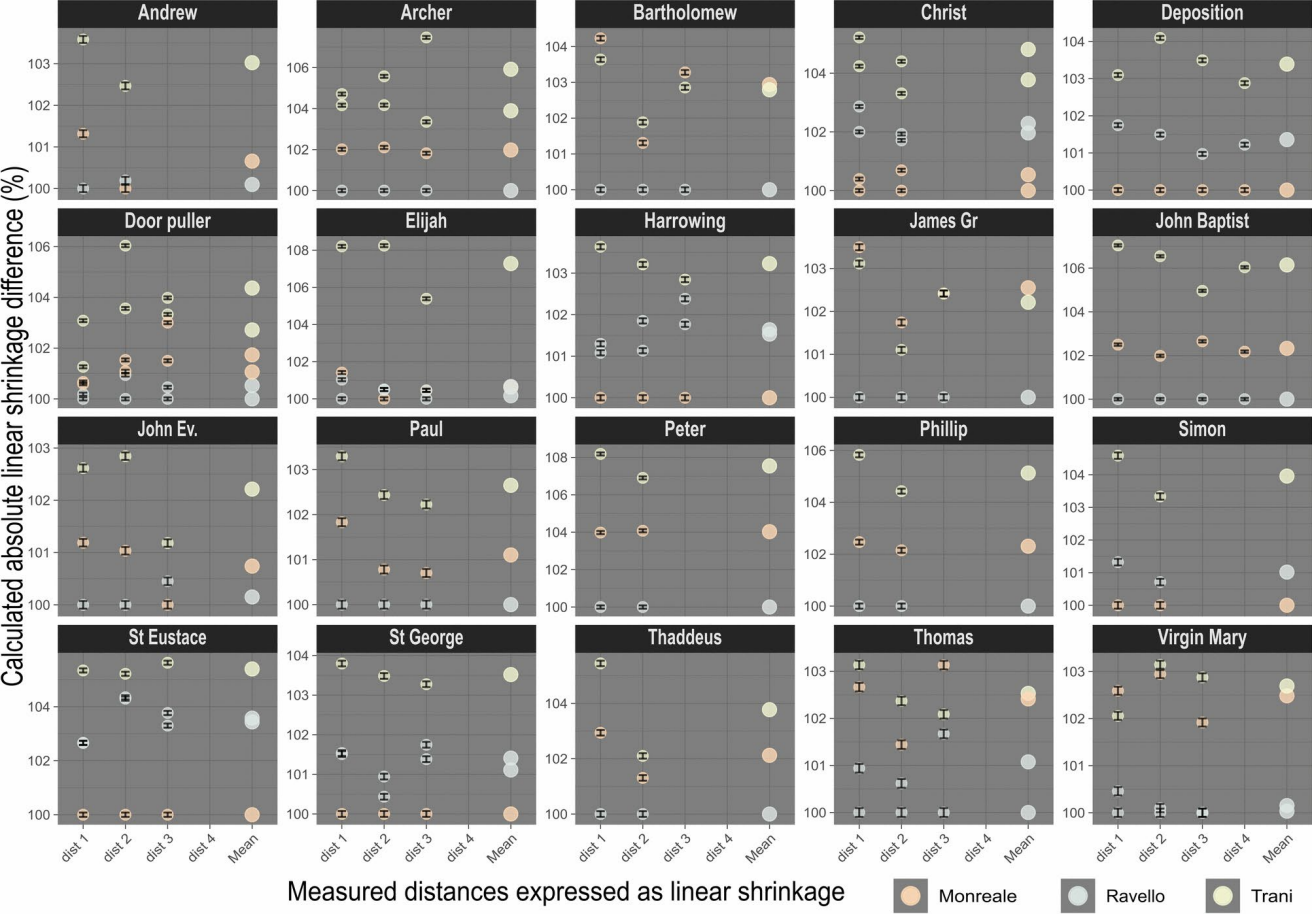
Panel shrinkage visualisation with errors and mean: All measurements concerning the panel shrinkage of all motif panels. Individual measurements (small dots) of each panel were expressed as mean value (larger dot) in relation to the smallest panel within any motif series. Propagated error is indicated as error bar.

Table 3 presents the measurements of 20 motifs being present in all three doors. By motif panels, we refer to the figurative relief depictions on the doors, explicitly excluding the surrounding friezes. In some cases, motifs appear on two panels on a single door. A total of 73 motif panels were measured, typically using three dimensions. Wherever possible, measurements were taken perpendicular to each other to capture the expected differences in the x and y directions (Fig. 6). However, this was not always feasible due to the absence of sufficiently distinctive features in some panels. Additionally, all the larger scroll work frieze panels were analysed as well (Table 4).

**Table 3:** All measurements that were taken on the motif panels of the three doors of Barisanus.

	Measured distances in mm						Measurement position
	Trani		Monreale		Ravello		
	Tr 1	Tr 2	Mo 1	Mo 2	Ra 1	Ra 2	
	A6		A5		D6	A6	St George
1	236.1		227.5		231.0	230.9	lance tip to dragon tail
2	142.6		137.8		139.1	138.4	hooves
3	141.9		137.4		139.8	139.3	horse tail to tip front hoof
	D6		D5		C6	F6	St Eustace
1	210.9		200.2		205.5	205.5	thumb-hoof bottom right
2	241.0		229.0		239.0	238.8	thumb to bottom left corner
3	104.3		98.8		102.0	102.5	tip ear to I
	D5		A2		E3		Deposition
1	129.8		125.9		128.1		Crossbeam incision top corners
2	216.0		207.5		210.6		Cross height right
3	233.9		226.0		228.2		Left corner crossbeam – tight ladder post bottom
4	235.7		229.1		231.9		Foot left corner – incision cross beam right top corner
	C1	B1	B1	C1	B1	E1	Christ Pantocrator
1	268.1	265.6	255.8	254.8	262.1	259.9	inner distance Mandorla
2	286.8	283.8	276.6	274.7	279.4	280.0	middle finger Jesus – corner right bottom Gospel St. Luke
	D3		B2		E2	B2	Harrowing of hell
1	239.5		231.1		234.1	233.6	ladder right foot post to cross left corner main beam
2	273.3		264.8		269.7	267.8	inside bottom left to top right corner
3	133.8		130.1		132.4	133.2	Crossbeam width at the top
	D2		D1		C5	F5	Elijah
1	199.1		186.6		185.9	184.0	right eye lid – big toe left
2	151.0		139.5		140.2	140.2	right thumb – hem right foot
3	115.4		110.0		110.0	109.5	bottom right hem to bottom left hem
	B4		D3		D4		Paul
1	141.1		139.1		136.6		Pillow width
2	105.2		103.5		102.7		width throne bottom
3	174.6		172.0		170.8		tip nose – left big toe
	C7	C6	C7		F7		Archer
1	211.2	210.1	205.8		201.7		arrow tip to right heel
2	225.4	222.4	218.0		213.5		tip hat to left toe
3	112.2	107.9	106.3		104.4		left toe- right heel
	A4		A1		A5		John the Baptist
1	167.2		160.1		156.2		Calve left -coat left- upper thumb
2	214.9		205.7		201.7		nimbus right frame- heel right foot
3	186.2		182.1		177.4		Index finger right frame – left foot small toe
4	175.6		169.2		165.6		Nose to right ball of the foot
	B2		C2		E5	B5	Virgin and Child
1	133.9		134.6		131.8	131.2	Pillow width
2	206.6		206.2		200.3	200.5	Headscarf edge forehead – left post corner left bottom
3	107.3		106.3		104.3	104.3	Distance post to post bottom corner
	D4		B4		C3		Andrew
1	141.6		138.5		136.7		Pillow width
2	211.8		206.7		207.1		Bottom right throne corner to cross tip right
	B3		A6		A3	D5	Thomas
1	131.6		131.0		128.8	127.6	Pillow width
2	198.7		196.9		195.3	194.1	T right top corner to bottom above incision right throne post
3	97.8		98.8		97.4	95.8	left incision left foot throne to right incision right foot throne bottom
	B5		A4		A4		Bartholomew
1	139.6		140.4		134.7		Pillow width
2	194.6		193.5		191.0		Top right eye lid right to bottom left big toe
3	219.6		220.5		213.5		bottom right big toe right eye lid
	A3		A3		A2		John Evangelist
1	129.5		127.7		126.2		top pillow width
2	198.9		195.4		193.4		right eye lid to bottom left foot throne
3	179.8		177.7		178.5		Big toe left to nose tip
	A2		D4		C2		James the Greater
1	135.7		136.2		131.6		Pillow width
2	174.0		175.1		172.1		Nose tip to bottom incision bottom right foot throne
3	190.7		190.7		186.2		left small toe to inner left eye lid
	C2		C3		C4		Peter
1	146.7		141.0		135.6		Pillow width
2	230.7		224.6		215.8		Bottom left corner post throne to top cross centre
	A5		C4		D3		Phillip
1	141.6		137.1		133.8		Pillow width
2	160.3		156.8		153.5		book top left corner to left post corner
	C3		D2		C6		Simon
1	118.6		114.9		113.4		Pillow width
2	189.0		184.2		182.9		Neck robe joint right
	C4		D6		F2		Thaddeus
1	122.0		119.1		115.7		Pillow width
2	179.8		178.4		176.1		right outer eye lid to right bottom corner

A measurement inaccuracy of ± 0.2 was identified by re-measuring panels.

**Table 4:** These measurements compare Type 1 frieze dimensions.

Door	Row	Left wing	Right wing
		in mm	in mm
Trani	1	287.1	287.1
Trani	2	287.6	282.5
Trani	3	288.7	280.4
Trani	4	288.7	288.8
Trani	5	287.6	285.6
Trani	6	286.6	284.0
Trani	7	289.5	285.7
Monreale	1	275.9	284.5
Monreale	2	277.8	278.8
Monreale	3	279.7	276.7
Monreale	4	277.1	280.5
Monreale	5	282.5	282.3
Monreale	6	277.7	280.9
Monreale	7	284.3	280.1
Monreale	8	278.3	285.3

They were taken from the tip of the bottom leaf to the tip of the bottom leaf in the case of Ravello and to the top leaf below the red line (Fig. 5) to ensure comparability. Manual measuring error is ± 0,2 mm to calculate the error propagation, which led to a relative propagated error of ± 0.1% in all cases.

The measurements demonstrate considerable differences in the dimension of otherwise identical motif panels. The recorded shrinkage between all the panels reaches a maximum of 8.2%, but is generally in the area of approximately 2.5%. A measuring error ± 0.2 mm was observed during the manual measuring of the panels and used for the calculation of the error propagation. Additionally, we observe a slightly non-uniform trend in the measurements, which cannot be further examined within the scope of this paper. However, these variations are thought to represent distortions resulting from the model’s replication process.

**Fig. 7:**
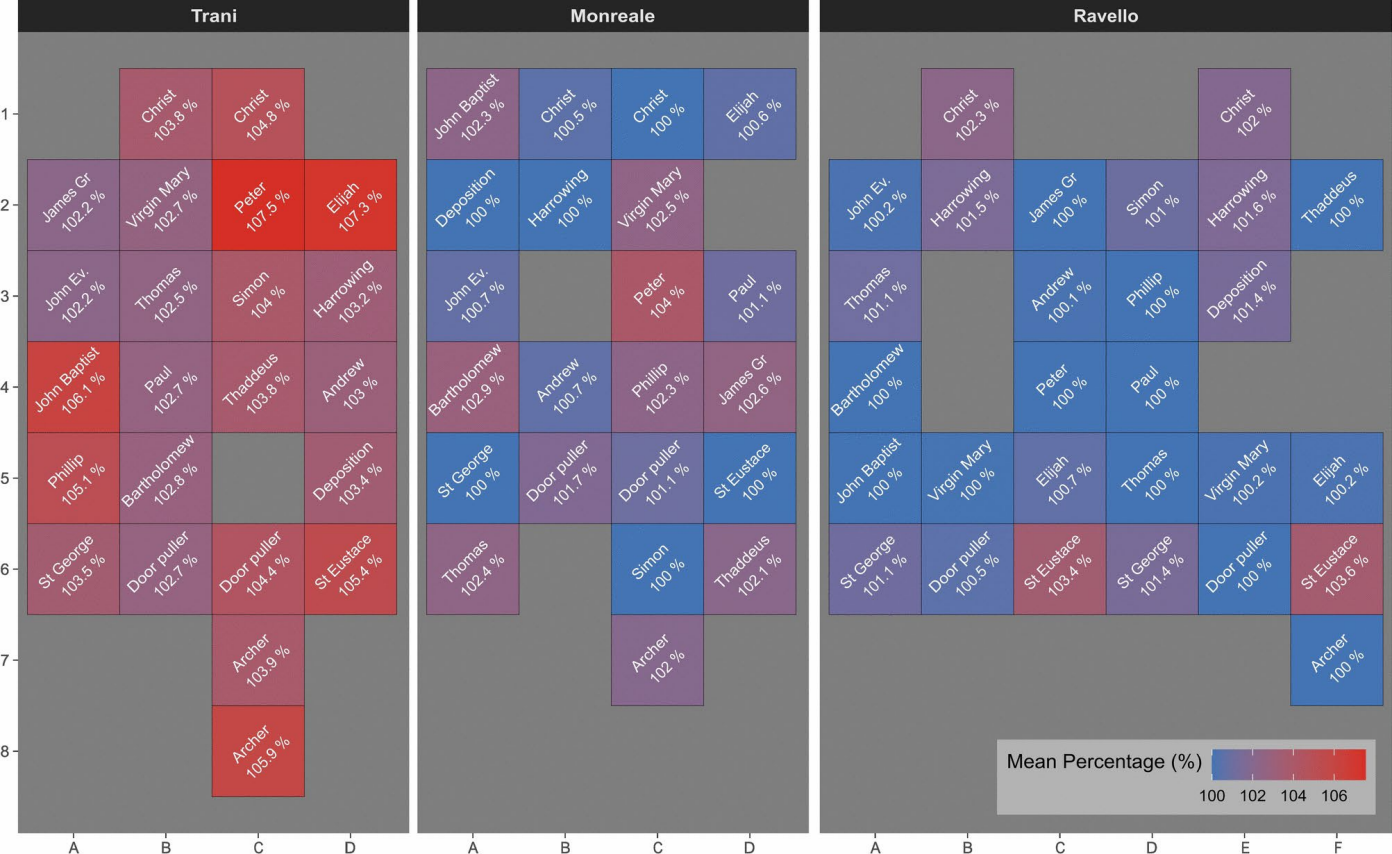
Heatmap visualisation of the absolute difference in dimensions. All measurements expressed as absolute difference in dimension across all doors and motif panels, that are present in all three doors.

In 16 out of the 20 panels included in the measurements, the motif panels from Trani are the largest, indicating that they represent a first casting generation among the three doors (Fig. 7). The remaining two panels, depicting Bartholomew and James the Greater, are from the Monreale doors and are only slightly smaller than those from Trani. Another two panels, Virgin Mary and Thomas, show similarly small differences in dimensions, though in these cases, the Monreale doors have the slightly larger motif panels. Given the minimal size differences in these four panels, it is reasonable to assume that they were made from the same model, and that the dimensional variation resulted from a replication process. This sheds light on the variability to be expected within the same casting generation.

To further explore this variability, we examined motif panels that appear twice on individual doors: Christ Pantokrator, the door pullers, St. George, St. Eustace, the Harrowing of Hell and Thomas. Though each door features these motifs twice, the dimensional differences between the two motifs vary from one to another: The difference between two panels in Trani is on average 1.4%, being close to the linear shrinkage of bronze, where in Monreale the mean is at 0.82% and in Ravello at 0.38%. Ravello possesses the largest number of duplicate motifs. The difference in the dimensions of the motif panels cannot be attributed to metal shrinkage in the cases of Monreale and Ravello. For the Trani doors this remains unclear. In Trani the larger difference could be attributed to metal shrinkage and the production of a second casting generation.

The analyses identify varying degrees of shrinkage. Recognizing 1.5% as an unavoidable linear shrinkage, numerous motifs exhibit values below this threshold, warranting further explanation. One important finding is that the copies within the Ravello doors are virtually identical in dimensions, suggesting that the replication practices used here may have been slightly more advanced.

As a rule in this smaller shrinkage is area of 1 ± 0.5%, which is what we interpret to be the result of the replication process of wax models. Based on this we estimate a combined mean shrinkage of 2.5 ± 0,5% between different casting generations of the motifs.

**Fig. 8:**
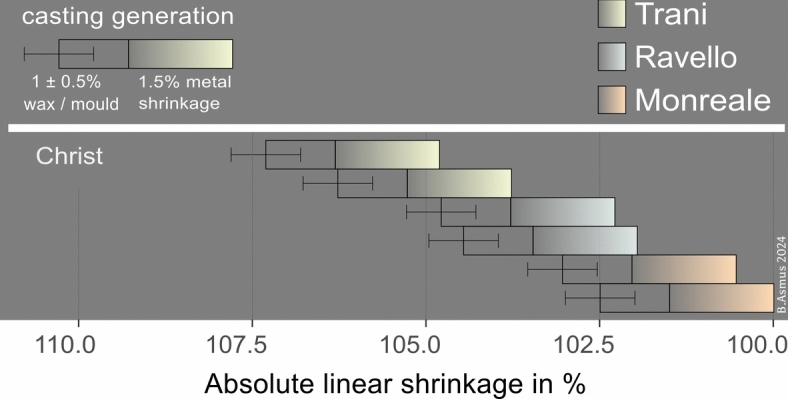
Linear shrinkage. A casting generation is defined by an estimated total linear shrinkage of 2.5 ± 0.5%, reflecting the combined shrinkage of wax/mould and metal. Dimensional differences were expressed as percentage relative to the smallest dimension of each motif series (Table 4).

Figure 8 introduces the concept of casting generations. The bars represent a complete casting generation and provide a visual comparison of dimensional differences between panels. The right end of each bar indicates the final dimension of the cast after cooling. Each bar is divided into two sections: First, the gradient-filled rectangle represents the solid linear shrinkage of 1.5% associated with the cooling and solidification of the metal. Second, the empty rectangle corresponds to the wax model’s final dimension on the right and the mother model or mould’s initial dimension on the left, accounting for an additional 1 ± 0.5%. Altogether, the total bar length represents a linear dimensional change of 2.5 ± 0.5%, combining both wax and metal shrinkage.

If the bars overlap, it suggests that the panels were not cast directly from one another. A minimum difference of 1.5%—the linear shrinkage of a copper alloy—is required to confirm that a piece was metal-cast from a model. Differences smaller than this threshold are more likely explained by the model being reproduced by other methods, such as remaking it in wax or pressing it directly into moulding clay.

The bars are arranged by their measured values, with colours indicating the door to which each panel belongs. On the left, the bars depict the dimensions of the mould in its initial, hot state. On the right, they reflect the actual dimensions of the cooled panels at room temperature.

**Fig. 9:**
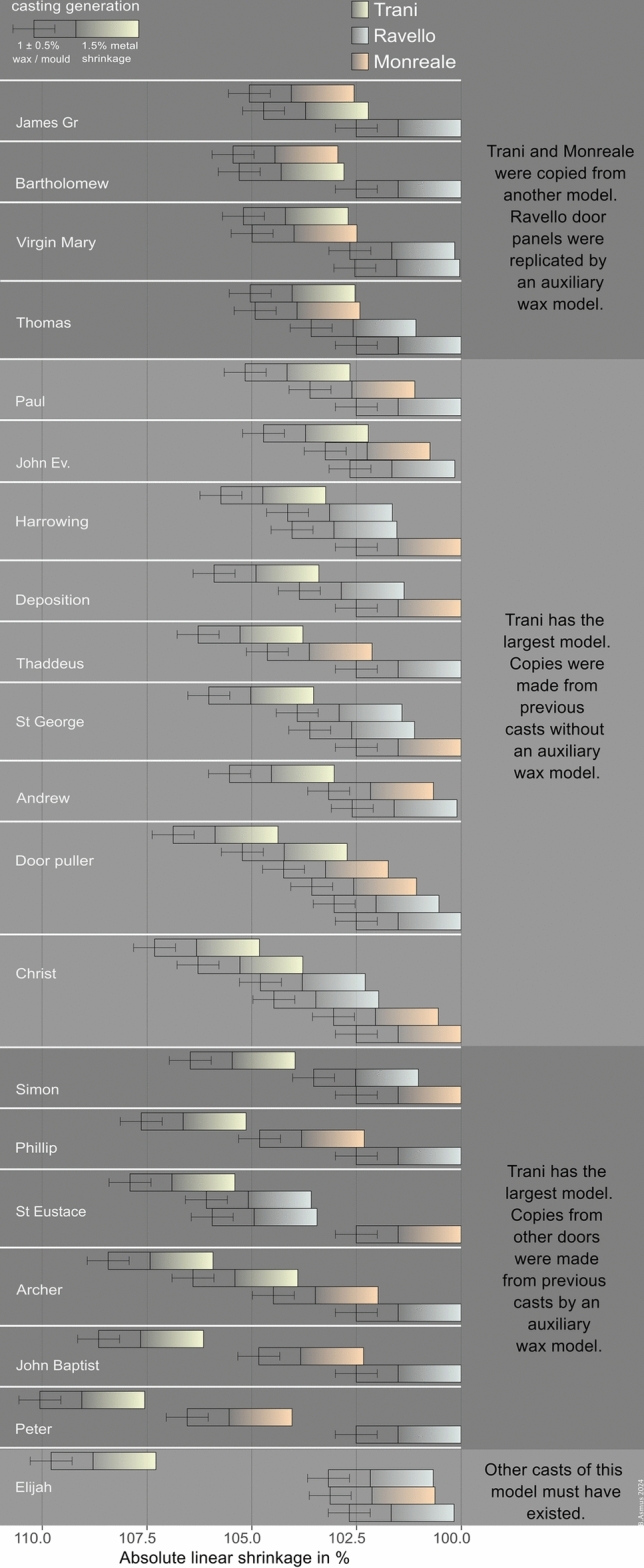
Casting generations. The plot compares the different shrinkage across all panels, and exhibits distinct patterns of shrinkages.

Figure 9 presents all examined panels. The panels are ordered according to their shrinkage values from top to bottom. Several aspects emerge:

1. In 16 panels it is beyond doubt that Trani is the “mother model” of the three analysed doors.
2. Four of those panels: Thomas, James the Greater, Virgin Mary, and Bartholomew this is not so. Another model must predate these, because Trani and Monreale are clearly reproduced from the same models.
3. The largest group of panels exhibits shrinkage values ranging from 1.5 to 2.5% between the doors, with differences of ~ 0.5–1% often observed within a single door.
4. A group of six panels, Simon, Phillip, St Eustace, Archer, John the Baptist, Peter, exhibits shrinkage values of 2.5% or more between the three doors. It is probable that these were copied from the metal casts by means of intermediate mould.
5. The Elijah panel of the Trani doors was probably not copied **directly** from Trani, but from another copy that does not exist any more.
6. There is no clear chronology of Ravello and Monreale. In Monreale six panels are among the smallest panels of a series. In Ravello there are fourteen of the smallest ones. It is quite probable that the panel moulds of those doors were made contemporarily.

To conclude: Within the framework of these three doors the Trani doors possess only panels from the first casting generation, so in relative terms, this door is composed of the oldest panels of these three doors. Monreale doors also possess two panels of the first casting generation, and otherwise motif panels with shrinkage values similar to those of Ravello. There is no statistically significant difference between the distribution of shrinkages in Monreale and Ravello. There is, however, a larger variability of shrinkages in Monreale. Especially a few first generation castings are present in Monreale, while not in Ravello. The doors from Trani possess undoubtedly the largest amount of first generation castings and should therefore be the doors that were created first, while the Monreale and Ravello panels were made contemporaneously.

### Friezes, scrollwork and composite friezes

**Fig. 10:**
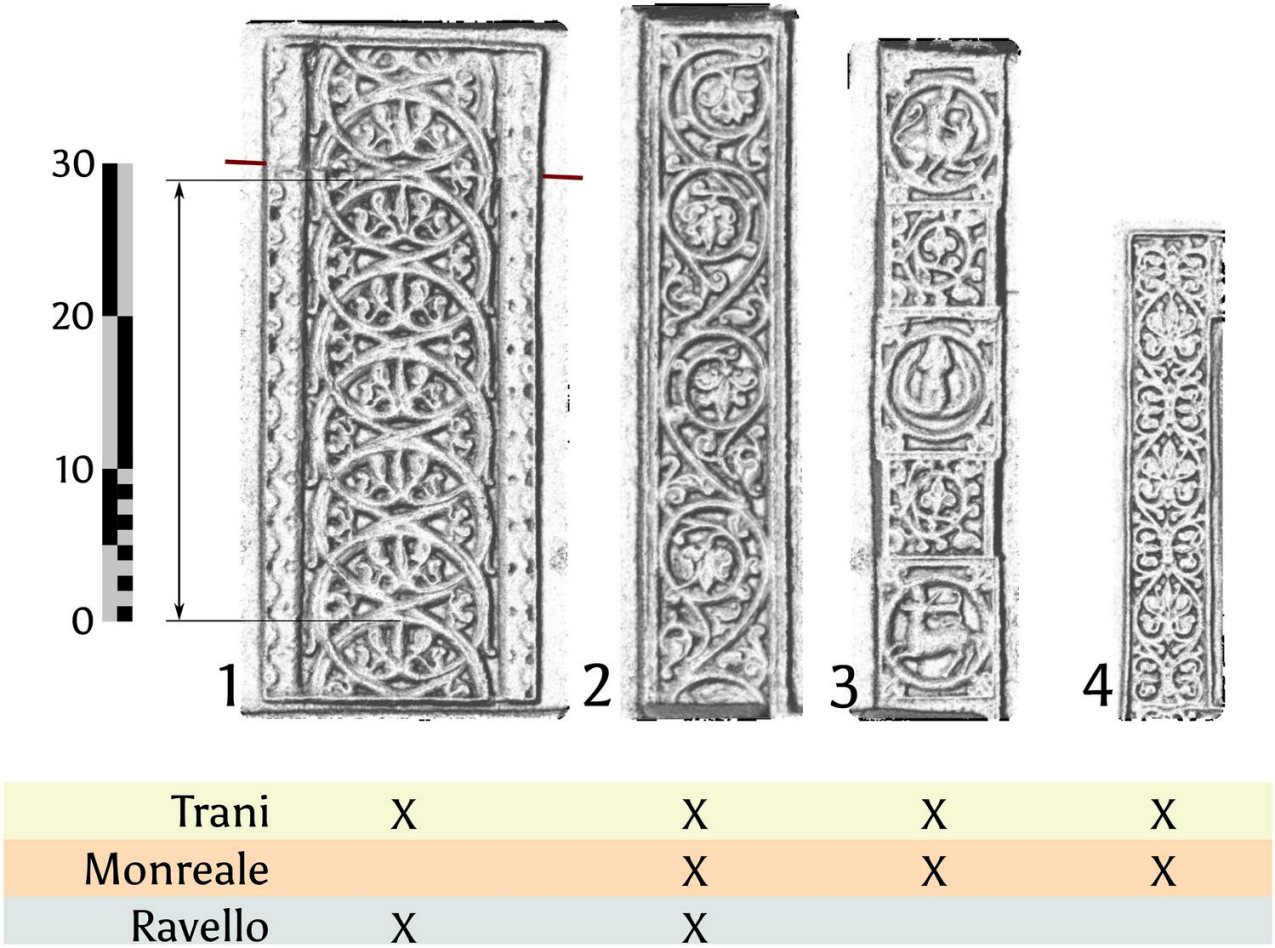
Frieze type shared across Barisanus’ doors. Note the below indication on which frieze is present in which door. The red line shows where the frieze is “pieced together” for Trani. The arrow shows where the measurements for Table 3 were taken. Detail images are extracted from a relief visualization in GIS, generated using a blended combination of surface raster calculations (raster resolution 0.2 mm), that highlights minute details of the relief. Image: B. Asmus based on M.Fera/Novetus/Gapamet licensed on CC-BY^1^.

Observing the extensive use of decorative friezes that Barisanus employed to frame the panels, along with the depicted biblical scenes and figures, it appears likely that these elements were created using a more advanced replication technique than what have been observed in many other European bronze doors. It does suggest that the techniques used for the Trani door were more sophisticated than those typically seen in comparable works. Scholars have proposed two main hypotheses: the use of stamps pressed into sand moulds^24^ and the use of auxiliary moulds to pre-fabricate wax models^2^. The Trani door shows four sets of friezes, which were used individually to produce the rather complex models for the Trani doors (Fig. 10).

There are four types of friezes:

1. Wide circular wine scroll friezes left and right of the motif panels set in their own panels;
2. Smaller scrollwork friezes that frame the complete door leaf in Trani and frame the panels in Ravello;
3. Compound friezes consisting of scrollwork and emblems of various biblical and mythological creatures;
4. Smallest rectangular scrollwork friezes that delineate the pictorial scenes directly.

The Trani doors feature a wider variety of friezes compared to the Ravello and Monreale doors, with distinct differences in size and composition. The latter two doors each possess a differing subset of the friezes present in the Trani doors. Monreale possesses three types of friezes (Type 2, 3, and 4), Ravello only two (Type 1 and 2), but an additional one not present in the other two doors. There is not one exact copy of any complete panel in any of the doors. This has some implications: The Trani doors possess the largest of the wide circular wine scroll friezes (Type 1), making it at least one casting generation older than the friezes present in Ravello.

**Fig. 11:**
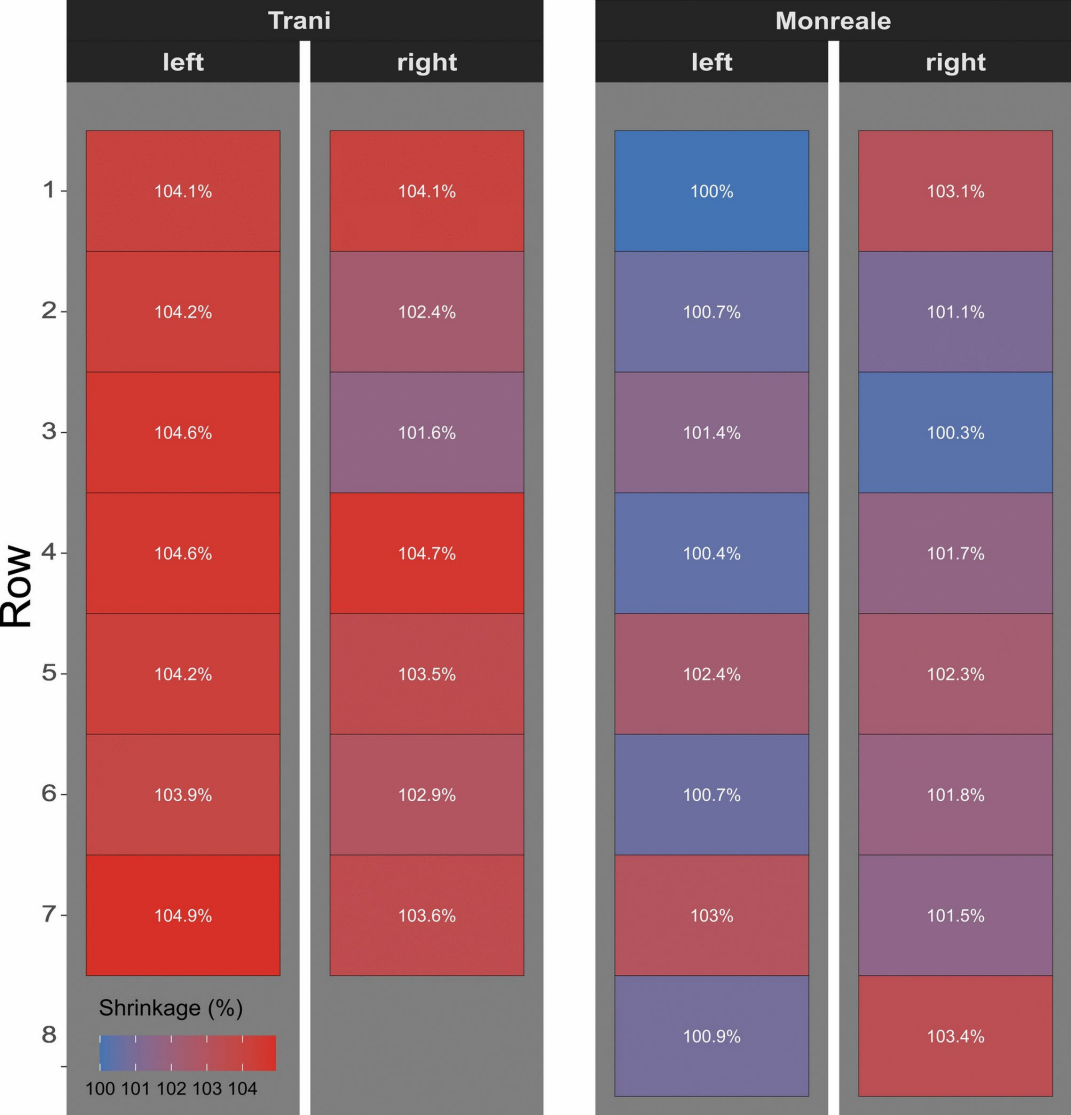
A heatmap displaying the absolute difference in dimensions of the Type 1 friezes from Trani and Monreale. The top left frieze of Monreale is the smallest, the bottom left frieze of Trani the largest cast object.

Four of the Trani type 1 scrollwork friezes are equal in size, the other four are 1%, 2% or 3% smaller (Table 4**, **Fig. 11). Type 1 frieze from the Ravello doors are smaller still and display shrinkage of 2–5%. Further, the friezes in Trani are pieced together to adjust them to the greater length needed for panel height in Trani. More important than the fact that this particular Trani frieze is pieced together, is the fact that these basic frieze units could apparently made either with relative ease or at least it was possible to produce them in sufficient quantity, because it is clear that these were used in a modular fashion to create **customised metal casting moulds or wax models**. We will demonstrate this below exemplarily.

**Fig. 12:**
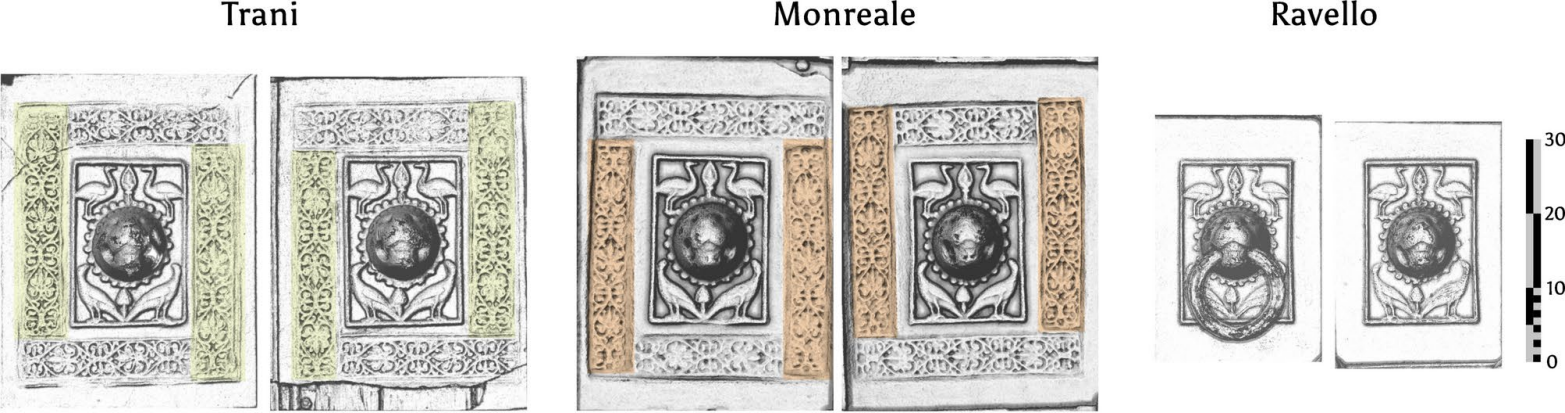
Door pullers exemplify the different dimensions of the panels, and the distinct composition of each panel with regards to the friezes. The Ravello doors do not even possess these friezes, so these are clearly not complete copies, but composites of copied modules. Detail image based on relief visualisation. Image: B. Asmus based on M.Fera/Novetus/Gapamet licensed under CC-BY^26^.

Looking at the panels containing ornamental door pullers at Monreale and Trani, we observe that the matter of arranging the scrollwork friezes around the door pullers differs from one door puller to the other (Fig. 12). If all six door pullers display distinct characteristics in the arrangement of their various parts, it is clear that they could not have been created using the same door puller panel model. Instead each model was made individually by using prefabricated pieces. These pre-fabricated frieze pieces share identical characteristics, dimensionally, as well as with regards to their physical traits. For this to work it is mandatory that a mechanical method of replicating these pieces existed. For the door pullers it is beyond doubt that the lost wax technique was used, because the casting moulds for these are very difficult to produce otherwise.

The right Trani door puller has the largest dimensions and is therefore most probably the model for the left door puller.

However, for all the other panels there is still another hypothesis to discuss. Without archaeological or historical evidence of such auxiliary moulds, the question is whether the individual metal casting moulds were created by pressing a positive die into the mould material or by using the lost wax technique.

### Macro traces in the panels: reverse sides, gating system, blurriness, casting defects

**Fig. 13:**
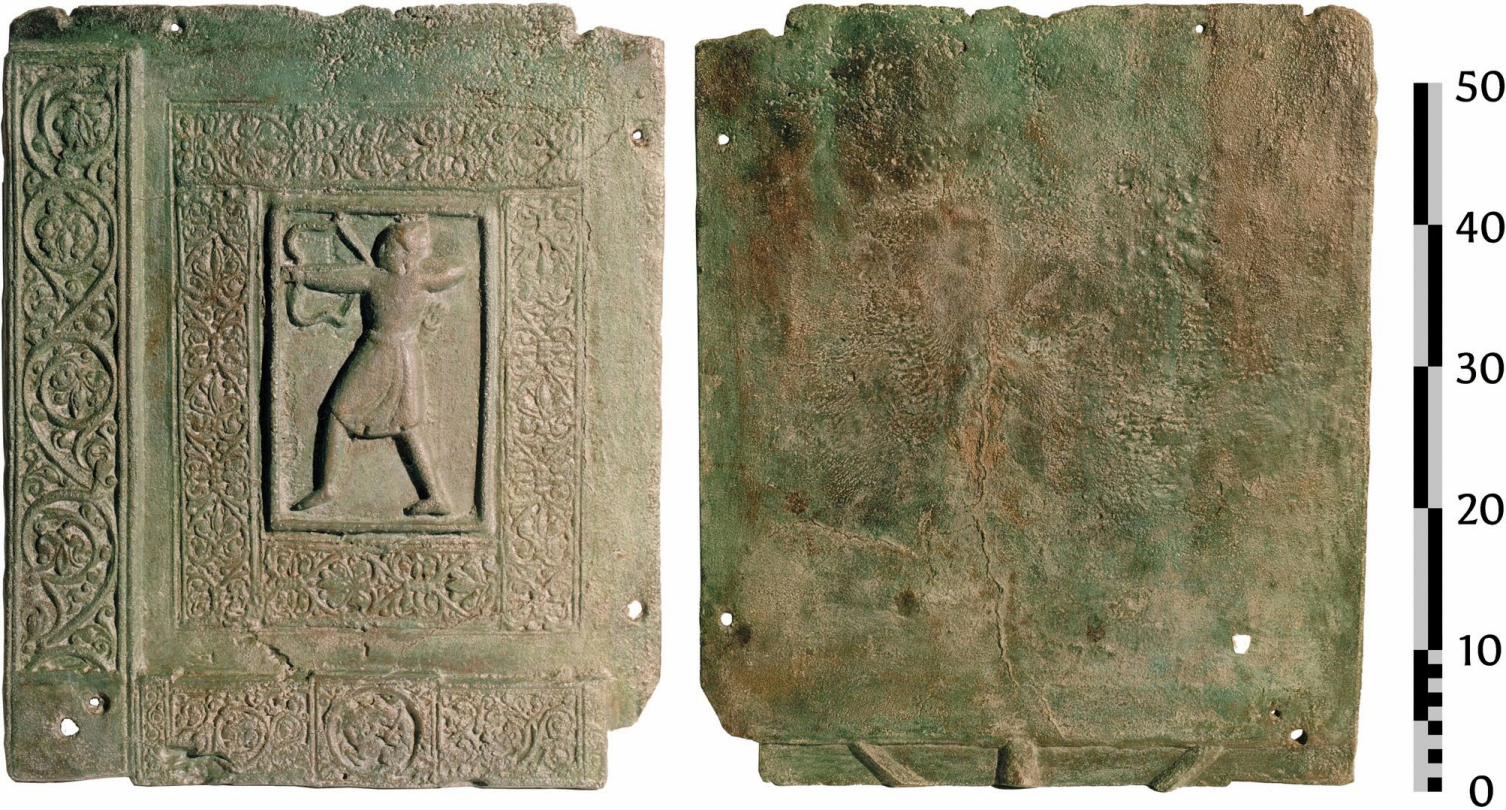
Trani, Italy. Door of Barisano, panel C8. This panel was overlapped by panels C7 (above) and D8 (to the right); frontside (left) and backside (right). The panel was cast upside down in a vertical manner. Reproduction with the authorisation of the Soprintendenza Archeologia, Belle Arti e Paesaggio per la città metropolitana di Bari—MiC. Any reproduction of this image is forbidden by the Soprintendenza.

The Trani doors provide further evidence for the casting process, particularly in Panel C8, an archer (Fig. 13).The back of this panel exhibits remnants of the gating system. The panel was cast vertically, with the motif upside down during casting. Unlike in lost wax casting, where the gating system can be freely designed and attached using wax rods in three-dimensional space, sand casting confines the gating system to the two-dimensional planes of the mould. In this case, the gating system seems to have been cut into the backside of the mould, a method familiar to those experienced with sand moulding.

When comparing the reverse sides of the Trani door, the distinctive style of the gating system immediately evokes parallels with the bronze doors of Augsburg, where a similar system is visible in 24 of the 35 panels^25^. This resemblance strongly recalls the way gating systems are shaped in modern sand moulding processes, particularly in art bronze casting (Asmus, personal observation).

Although we are sceptical of a “sand mould” hypothesis, it is highly plausible that a method was employed that left traces bearing striking similarities to those produced by sand moulds. The main problem is with the scientific approach: a modern industrial moulding process is projected onto heritage objects, thereby omitting to understand the objects production in the light of their regional, historical and technological traditions. There is no evidence for large casting frames. Casting frames of 80 cm by 80 cm are necessary to cast these panels. Furthermore, as laid out above, evidence of sand-casting purportedly written by al-Jazari^17^ is not conclusive, and warrants at the very least a new critical translation of the sections in question.

The authors support that the technique in question is, in many respects, a direct precursor to modern sand moulding. Specifically, it likely involved a two-part or multi-part mould composed of casting loam. This method would have allowed for the retrieval of a semi-/permanent model used as a positive die, or alternatively, a more intricate model that was sacrificed through molten removal when its complete extraction was impractical.

A similar hypothesis was partly formulated by Lüer^4^, though it seems to have been mostly overlooked in subsequent studies.Even more compelling, and potentially decisive in this debate, is Brendels observation that the image panels exhibit an offset and remnants of a flash-line^25^. This provides unequivocal evidence for the use of two-part moulds. While cited in connection with the Augsburg doors^25^, Lüer’s observation was far more general in nature. His statements, though interesting, were often presented as assertions rather than being rigorously proven or critically discussed in the light of historical context^26^, or considerations of applicability in a medieval foundry context. Like many early textbooks on the subject, his work carries an air of authority that often substitutes for detailed analysis, leaving key questions unexplored. The key questions primarily focus on how the development of casting technologies can be understood within the technological scope of their time—considering not only the available methods and materials but also the prevailing paradigms that shaped artisans’ approaches and limitations. In this sense, it is not enough to apply modern engineering concepts or early 20th-century art foundry processes and project them onto the medieval period, as this risks overlooking the unique technological mindset and constraints of the time.

Of note is also the completely flat surface on the back, making it less likely that this particular panel was pieced together from individual wax models, but rather was produced by impressing several models into the green moulding material. In this scenario only the front is moulded, the back half could have been made from a flat piece of moulding loam.

### Blurriness

Cast objects can appear blurred because of a number of reasons. Areas that are accessible to the public will result in a partial polishing of prominent features such as heads, noses, etc. It may lead to a complete loss of surface features. Blurriness can be a function of the metallostatic pressure during casting. In these cases, the regions with the lowest pressure will result in unsharp motifs. These traces can be used to identify the orientation of the motif during casting. The third factor that may contribute towards a blurry appearance is residual moisture in the moulds, caused by insufficient drying or baking of the mould. During casting, a vapour pillow prevents exact mould filling. The moisture content of a mould may result in a whole spectrum of casting defects, with the blurriness being one of the least problematic ones.

### Process hypothesis of the casting technology

Based on the traces observed on the cast objects, and particularly on the shrinkage values, a more detailed understanding of the casting and reproduction processes emerges. We propose the following modes of reproduction:

The reproduction of moulds was achieved through at least two methods:

1. **Stamping or Impressing with a permanent model**: Producing a mould by pressing the model into the metal casting mould material.
2. **Intermediate/auxiliary mould**: A model was first reproduced using an auxiliary mould and the mould was made thereafter by the lost wax technique, or by using it to impress this into the mould material.

Each method offered distinct advantages for workshop practices, and are especially applicable when working with low relief objects, such as the majority of the door panels. If the workshop already possessed the model, a metal casting mould could be easily produced by stamping or impressing the model into the mould material. The resulting shrinkage from this approach is limited to the metal, provided the artisan was skilled in procuring suitable casting loam. If the model was not available in the workshop, an alternative approach could be employed. The model could be cast from an existing door by using fine ball clay to create an intermediate wax model. After retouching, the wax model could either be used to produce a lost wax mould, or to create an impression in the casting mould material.

The examination of the cast pieces and their production traces clearly shows that a simplistic classification as being cast using the lost-wax process or the sand-casting method falls short of reflecting the realities of medieval foundry practices. It fails to encompass the range of possibilities that the three raw materials wax, clay and sand have to offer. They can be made into a sand mould, they can be made into a lost wax mould, they also can be made into a fired model of clay that is being coated in a thin layer of wax as a releasing agent. If the raw material base is expanded, so are the possibilities to make casting moulds, replication moulds, stamps or models. So, not only is this categorization far too narrow, but it also underscores the necessity of examining medieval foundry products in much greater detail, particularly with regard to the workshop practices and technological capabilities of the time.

## Conclusions

Based on detailed measurements of the orthophotos, we quantified the amount of shrinkage using all motif panels that are present in Barisanus’ doors at Monreale, Trani, and Ravello. While there are more panels than those we measured, our analysis included all motif panels common to all three doors. This led to a sequence of casting generations, where one cast likely served as a model for the next generation of casts. At least three, possibly five casting generations, could be identified. Consequently, a chronological order of the casting of the motif panels could be established and a sequence of production of the three doors proposed: Trani, Monreale and, more or less contemporary with the latter, also Ravello. Analyses suggest the existence of another door produced prior to the Trani door, possibly the door from Bari. This inference arises from the presence of four panels lacking definitive prototype models and a fifth panel exhibiting excessive shrinkage. The degree of shrinkage observed cannot be accounted for by a single casting generation between the Trani door and the other doors, indicating at least one additional intermediate casting generation, between Trani and Ravello/Monreale, as well between Trani and one prior door.

Furthermore, we could show that despite the lack of archaeological evidence of any auxiliary moulds or dies, a well established means of mechanical replication must have been known to the workshops that produced these monumental bronze doors. Especially the multitude of recurring use of various frieze types in all of the doors are proof for this.

The panels in these doors are distinctly individual in the manner of the arrangement of recurring motifs and friezes. They demonstrate that the models/moulds to create the panels were pieced together for every single panel. Within the analysed panels there is not a single case of a 1:1 copy of a complete panel.

As an afterthought and although this is difficult to prove, it seems unlikely that the doors of Ravello and Monreale were produced locally. The similarities in dimensions are too close between those two doors, and the chronology of their panel making is too simultaneous, so that it seems very likely that these two doors were made by the same workshop, respectively by artisans who had access to these mould, models or panels, and were then shipped off in parts to their destinations.

## Data Availability

All data generated or analysed during this study are included in this published article.

## References

[1] Mödlinger, M., Asmus, B., Fera, M., Utz, J. & Ghiara, G. The 12th century bronze doors of Barisanus of Trani in Trani, Ravello and Monreale. *PLOS ONE*. 10.1371/journal.pone.0319697 (2025). 10.1371/journal.pone.0319697 PMC11940662 40138380

[2] Mende, U. *Die Bronzetüren des Mittelalters : 800 - 1200*. (München, 1983).

[3] *Die antiken Grossbronzen. Bd. 1: Die antike Erzgestaltung und ihre technischen Grundlagen / von Kurt Kluge*. (W. de Gruyter & Co, Berlin, 1927).

[4] Lüer, H. *Technik der Bronzeplastik* (H. Seemann, 1902).

[5] Ristow, S. & Steiniger, D. Forschungen an den Bronzen des Aachener Domes. *Kölner und Bonner Archaeologica* **6**, 143–168 (2016).

[6] Ristow, S. Archäologie des Aachener Domes zwischen spätantiker und ottonischer Zeit (400–1000). In *Die Aachener Marienkirche: Aspekte ihrer Archäologie und frühen Geschichte* (eds Müller, H. et al.) (Schnell & Steiner, 2014).

[7] Pohle, F. *Die Erforschung der karolingischen Pfalz Aachen: zweihundert Jahre archäologische und bauhistorische Untersuchungen* (Verlag Philipp von Zabern, 2015).

[8] Buccolieri, G. et al. Non-destructive in situ investigation of the study of a medieval copper alloy door in Canosa di Puglia (Southern Italy). *Heritage* **5**, 145–156 (2022).

[9] Buccolieri, G. et al. In situ investigation of the medieval copper alloy door in troia (Southern Italy). *Heritage* **6**, 2688–2700 (2023).

[10] Brepohl, E. *Theophilus Presbyter und das mittelalterliche Kunsthandwerk. Band 2 Goldschmiedekunst*. (Böhlau, 1999).

[11] Asmus, B. Theophilus und der Guss einer Bienenkorbglocke. *Ein Experiment. Der Anschnitt* **68**, 45–60 (2016).

[12] Best, G., Halekotte, T. & Asmus, B. Eine Theophilusglocke aus dem Jahre 2018 - Über den Guß für die Bartholomäuskapelle in Paderborn und ihre beiden ‘Zwillingsschwestern’ aus und in Westfalen. in *Grammaticus - Praeceptor Translogiae - Artis Musicae Peritus - Investigator Sonitus Campanarum. Festschrift für Rüdiger Pfeiffer-Rupp zur Vollendung des 70. Lebensjahres* 1–30 (Gescher, Westf., 2019).

[13] Biringuccio, V. *The Pirotechnia of Vannoccio Biringuccio: A Classic Sixteenth-Century Treatise on Metals and Metallurgy / Translated from the Italian with an Introduction and Notes of Cyril Stanley Smith and Martha Teach Gnudi* (Dover Publications, 1990).

[14] Dines, I. The Theophilus Manuscript Tradition Reconsidered in the Light of New Manuscript Discoveries. In *Zwischen Kunsthandwerk und Kunst: Die Schedula diversarum artium* (eds Mauiège, M. & Westerman-Angerhausen, H.) (de Gruyter, 2014).

[15] Ilg, A. *Theophilus Presbyter. Schedula Diversarum Artium.* vol. 7 (Wilhelm Braumüller, Wien, 1874).

[16] Freise, E. Roger von Helmarshausen in seiner monastischen Umwelt. *Frühmittelalterliche Studien* **15**, 180–293 (1981).

[17] al-Jazari, I. al-Razzāz. *The Book of Knowledge of Ingenious Mechanical Devices. Donald R. Hill (Translator).* (D. Reidel, Boston, 1974).

[18] La Niece, S. Sand casting in the Islamic World. In *Verborgenes Wissen Innovation und Transformation feinschmiedetechnischer Entwicklungen im diachronen Vergleich* (eds Armbruster, B. et al.) 263–276 (Edition Topoi, 2016).

[19] Hirschfeld, Y. & Gutfeld, O. *Tiberias: Excavations in the House of the Bronzes. Final Report, Volume 1: Architecture, Stratigraphy and Small Finds.* (Jerusalem, 2008).

[20] Khamis, E. The Fatimid Metalwork Hoard from Tiberias: Tiberias: Excavations in the House of the Bronzes: Final Report, Volume Ii. *Qedem* **55**, III–428 (2013).

[21] Datenblätter – Kupferverband. https://kupfer.de/mediathek/datenblaetter/.

[22] Asmus, B. Bridging the past and present by skill: Exploring medieval bell casting by experiment. *Histor. Metall.* **54**, 85–102 (2024).

[23] Salmang, H. & Scholze, H. *Keramik* (Springer, 2007).

[24] Boeckler, A. Die Bronzetüren des Bonanus von Pisa und des Barisanus von Trani (Berlin 1953).

[25] Diemer, D. & Diemer, P. Die Augsburger Bronzetür. *Zeitschrift des deutschen Vereins für Kunstwissenschaften* **65**, 9–92 (2013).

[26] Mödlinger, M., Asmus, B. & Ghiara, G. The, “Schwarze Mander” of the Court Church in Innsbruck, Austria: Manufacture and Production of Monumental Brass Statues in the Renaissance. *Inter Metalcast*10.1007/s40962-024-01299-4 (2024). 10.1007/s40962-024-01299-4 PMC11824370 39949955

